# HBM4EU chromates study – the measurement of hexavalent and trivalent chromium in exhaled breath condensate samples from occupationally exposed workers across Europe^☆^

**DOI:** 10.1016/j.toxlet.2022.12.009

**Published:** 2023-02-15

**Authors:** Elizabeth Leese, Kate Jones, Beatrice Bocca, Radia Bousoumah, Argelia Castaño, Karen S Galea, Ivo Iavicoli, Marta Esteban López, Veruscka Leso, Sophie Ndaw, Simo P. Porras, Flavia Ruggieri, Paul T.J Scheepers, Tiina Santonen, HBM4EU chromates study team, Rob Anzion, Andrea Cattaneo, Domenico Maria Cavallo, Giuseppe De Palma, Giovanni Forte, Risto Lehtinen, Piero Lovreglio, Mathieu Melczer, Marta Senofonte, Sally Spankie, Maurice van Dael

**Affiliations:** i Radboud Institute for Biological and Environmental Science, Radboud University, Nijmegen, the Netherlands; j Department of Science and High Technology, University of Insubria, Como, Italy; k Department of Medical and Surgical Specialties, Radiological Sciences, and Public Health, University of Brescia, Brescia, Italy; l Istituto Superiore di Sanità, Rome, Italy; m Finnish Institute of Occupational Health, Helsinki, Finland; n Interdisciplinary Department of Medicine, University of Bari, Bari, Italy; o French National Research & Safety Institute, France; p Institute of Occupational Medicine (IOM), Edinburgh, EH14 4AP, UK; a Health & Safety Executive, Science and Research Centre, Harpur Hill, Buxton, Derbyshire SK17 9JN, UK; b Istituto Superiore di Sanità, Rome, Italy; c French National Research & Safety Institute, France; d National Centre for Environmental Health, Instituto de Salud Carlos III, Madrid, Spain; e Institute of Occupational Medicine (IOM), Edinburgh EH14 4AP, UK; f Department of Public Health, University of Naples Federico II, Naples, Italy; g Finnish Institute of Occupational Health, Helsinki, Finland; h Radboud Institute for Biological and Environmental Science, Radboud University, Nijmegen, the Netherlands

**Keywords:** Chromium speciation, EBC, Human biomonitoring, Welders, Chrome platers

## Abstract

The aim of this study was to investigate the practicability of exhaled breath condensate (EBC) as a biological matrix to detect and measure hexavalent chromium (Cr(VI)) and trivalent chromium (Cr(III)) in workers occupationally exposed to Cr(VI). EBC samples were collected from workers in France, Finland, Italy, The Netherlands and the United Kingdom from three different target activities: chrome platers, stainless steel welders and surface treatment workers. Pre and post working week EBC samples were collected from 177 exposed workers and 98 unexposed workers (control group). Hyphenated chromatography systems with inductively coupled plasma – mass spectrometry (ICP-MS) were for the analysis. The results showed that the occupationally exposed workers had significantly higher levels of Cr(VI) and Cr(III) than the control group. Chrome platers exhibited the highest Cr(VI) levels in their EBC samples, with a significant increase from their pre to post samples for both Cr(VI) and Cr(III). A significant difference was also found between pre and post EBC samples for Cr(III) in welders. This study has shown that EBC has the potential to be a valid, non-invasive biological matrix to assess occupational exposure to Cr(VI) and Cr(III) for biological monitoring assessment, with the ability to detect low level inhalation exposures.

## Highlights

• Exhaled breath condensate (EBC) reflects tri- and hexavalent chromium in the lungs.
• Both chromium species were higher in EBC of workers compared to controls.
• Both chromium species increased from pre to post workweek EBC in chrome platers.
• EBC may provide lung-specific, non-invasive occupational exposure data for hexavalent chromium.

## Graphical Abstract

**Figure:**
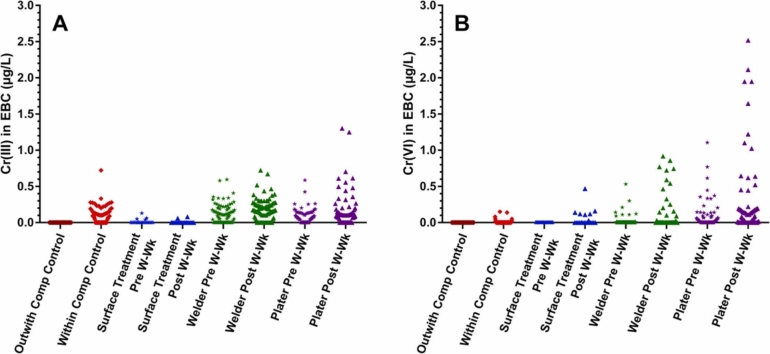

## 1 Introduction

The Human Biomonitoring for Europe (HBM4EU, www.hbm4eu.eu) initiative aims at improving chemical risk management and to provide support for policy making by understanding human exposure and the related health risks to chemicals in the environment, in occupational settings or by the use of consumer products (Santonen et al., 2019). With experts from 30 European countries involved in this project, a main focus is the harmonisation of methodologies and a standardised approach for data collection to enable comparisons of the findings and use of the data in risk assessments across Europe (Lopez et al., 2021).

One work strand within this project is investigating workers’ exposure to hazardous chemicals in the workplace. Hexavalent chromium (Cr(VI)) was identified as a chemical of concern by the HBM4EU programme (HBM4EU). A multi-centre study consisting of 9 different countries, namely, Belgium, Finland, France, Italy, Luxembourg, Poland, Portugal, The Netherlands and the United Kingdom (UK) explored occupational exposure to Cr(VI) to determine if current safety and control measures used in workplaces across Europe are sufficient for protecting workers. This was achieved by generating new occupational exposure data, investigating different biological matrices to test new methods of Cr(VI) exposure assessment, to compare Cr(VI) levels of the occupationally exposed and an unexposed working age population. The outline, study design and protocol of the HBM4EU chromates study was published by Santonen et al. (2019). This describes ethical approvals, worker recruitment, standard operating procedures (SOPs) and the collection of urine, blood and exhaled breath condensate samples and, to help understand the contribution of each route of exposure, personal air samples, hand wipes and relevant worker and workplace contextual information. In addition, Santonen et al. (2022) published a summary of the results encompassing all sample matrices collected from the whole of the HBM4EU chromates study. Herein, we present in detail one aspect of the HBM4EU chromates study, the investigation of the novel biological matrix exhaled breath condensate (EBC) and its usefulness in assessing occupational exposure to Cr(VI).

Chromium is an element whose various compounds exhibit different levels of toxicity. Trivalent chromium (Cr(III)) is less toxic and considered an essential nutritional element (Nordberg et al., 2007). Whereas, Cr(VI) compounds (chromates, chromium trioxide and dichromates) are classified by the International Agency for Research on Cancer (IARC) as carcinogenic to humans (Group I) (IARC, 2021). The health effects associated to Cr(VI) are extensive, with the route of exposure resulting in different health effects, including allergic contact dermatitis from skin contact (ATSDR, 2012, Barceloux, 1999) and respiratory effects such as ulceration of the nasal mucosa, occupational asthma and nasal, sinus and lung cancer (ATSDR, 2012, IARC, 2012, Rakhunde et al., 2012) from inhalation exposures. The types of industries and occupational activities where Cr(VI) exposure occurs are the chrome plating industry, other surface treatment industries (such as paint application and removal of old Cr(VI) containing paint), the production and use of stainless steel and other chrome alloys including welding, cutting and finishing and smelting (HSE, 2013, IARC, 1990, IARC, 2012). Although the route of exposure to Cr(VI) can be through skin absorption or ingestion, the primary route in the aforementioned industries is through inhalation of dusts, fumes and mists (IARC, 1990, OSHA, 2006) resulting in local respiratory tract effects, including cancer. However, no matter the exposure pathway, once within the body Cr(VI) is reduced to Cr(III) in all biological fluids, before being mostly eliminated in urine (De, 2000, De Flora et al., 1989; IARC, 2012). It is possible to measure chromium exposure in urine or red blood cells (RBC) (ATSDR, 2012, Flanagan et al., 2020). The measurement of RBC is only indicative of Cr(VI) exposure, measured as Cr(III) (due to Cr(VI) cell permeation and rapid sequential reduction from Cr(V) to Cr(IV) and finally Cr(III)) (De Flora et al., 1989; Hoet, 2005). However, due to some Cr(VI) being reduced in the plasma limiting RBC penetration (Hoet et al., 2005), the invasive nature of sampling and the need for trained medical staff to collect blood samples, neither employers nor employees favour providing a blood sample for biomonitoring of Cr(VI) exposure assessment (Cocker et al., 2007). Therefore, urine analysis is widely used, measuring total chromium concentration. This measurement of total chromium in urine is unable to distinguish between Cr(VI) and Cr(III), meaning Cr(VI) exposure cannot be specifically determined (Remy et al., 2021). This method of exposure measurement is, nonetheless widely accepted and, although not ideal in determining Cr(VI) exposure, it is a practical approach (ACGIH, 2021; Hoet, 2005; HSE, 2020; Remy et al., 2021). However, as workplace exposure limits reduce, methods to analyse the total chromium concentration in urine will become less useful as it will be difficult to separate the harmful Cr(VI) exposure from dietary Cr(III) exposure and occupational Cr(III) exposures. For example, in Europe, the binding Occupational Exposure Limit (OEL) for Cr(VI) is currently 0.01 mg/m^3^ (8-hour time weighted average (8-h TWA)), reducing to 0.005 mg/m^3^ 8-h TWA in 2025 (ECHA, 2021). Other countries (for example France and the Netherlands) have a much lower OEL of 0.001 mg/m^3^ for Cr(VI) (JORF, 2012, SER, 2016). In light of this, more specific biomarkers are being investigated, such as Cr(VI) in EBC.

It is thought that EBC might be a useful biological matrix to reflect inhalation exposure, and advancing the investigation of EBC may further the understanding of inhalation exposures and how elements behave and reside in the lungs. For Cr(VI), which causes lung diseases, it is also potentially a target-organ-relevant matrix and may give direct insight into tissue dose.

EBC is a biological fluid, consisting mainly of water vapour but also small droplets of airway lining fluid from within the bronchial and alveoli regions of the lungs. EBC can also contain droplets from the entire respiratory tract including the mouth and trachea (Hunt, 2007). Within these droplets of airway lining fluid is an unknown fraction of both volatile and non-volatile substances including environmental and occupational contaminants (Goldoni et al., 2005, Hunt, 2002).

Since EBC is free of interfering solutes found in other biological samples it makes it an ideal biological matrix for elemental analysis. This technique has previously demonstrated that occupationally inhaled elements can be detected in EBC, for chromium (Caglieri et al., 2006, Goldoni et al., 2006, Hoffmeyer et al., 2011, Leese et al., 2017, Riccelli et al., 2018), cobalt and tungsten (Broding et al., 2009, Goldoni et al., 2004), beryllium (Hulo et al., 2016), lead (Felix et al., 2015) and manganese (Hulo et al., 2014).

The purpose of the study reported here was to investigate the usefulness and applicability of EBC to detect and measure Cr(VI) and Cr(III) in workers occupationally exposed to Cr(VI). EBC samples were collected from workers in five of the nine countries participating in the HBM4EU chromates study from three different target activities: chrome platers, stainless steel welders and surface treatment workers.

The aims were:

1. To investigate EBC as a potential biological matrix for the exposure monitoring of Cr(VI).
2. To determine if speciation analysis of EBC can be an alternative sample matrix to urinary total chromium analysis in low level Cr(VI) exposures and/or where workplace exposure limits have been reduced making total chromium concentrations in urine less useful
3. To determine if there was a difference in Cr(VI) and Cr(III) between the different workers of the three target activities.

The wider HBM4EU chromates study collected urine, blood, air measurements and hand wipe samples, in addition to contextual information via a worker questionnaire (results not shown here but published by Santonen et al. (2022)).

## 2 Method & materials

### 2.1 Study population

EBC samples were collected from workers (n = 177) who were occupationally exposed to Cr(VI) via three target activities; chromium plating, surface treatment (painting or spraying) and stainless-steel welding. All were over the age of 18 years with a mean age of 41 years (at the time of recruitment). Samples were also collected from workers not involved in the three target activities to form a comparison control group (n = 98). All were over the age of 18 years, with a mean age of 44 years. The occupationally exposed group consisted of mainly men (99%), whereas in the control group it was approximately a 3:1 ratio of men / women (77% men v 23% women). Workers in the control group were split into two categories, those who worked within the same company as the occupationally exposed but were not involved in the target activities (Within Company controls) and those who worked in companies or industries separate from the target activities or any type of metal working (Outwith Company controls).

Ethical approval was obtained in each of the participating countries (Santonen et al., 2022). All participating volunteers gave written informed consent. All relevant documentation is in the supplementary material of Santonen et al. (2019).

### 2.2 Sample collection

A standardised approach to EBC sample collection was proposed to ensure the collection of harmonised comparable data from each participating country. An SOP (available in the supplementary material) was developed for the collection of EBC samples and transport to the analysing laboratories only (analysis was not included in this SOP). The SOP included the requirement of an EBC collection device by a single manufacturer to be used by all (as recommended by the American Thoracic Society and European Respiratory Society Task Force for comparing study data) (Horvath et al., 2005, Horvath et al., 2017). For both the occupationally exposed group and the control group, workplace site visits were conducted by a research team from each participating country.

For the occupationally exposed workers, two EBC samples were collected. A pre working-week sample, for example on a Monday morning at the start of the shift, and a post working-week sample taken towards the end of the week, for example a Thursday afternoon at the end of the shift. For the comparison control group only one EBC sample was collected, at any time during the working day.

All EBC samples were collected using a portable Turbo-DECCS condenser (Transportable Unit for Research on Biomarkers Obtained from Disposable Exhaled Condensate Collection Systems) by Medivac (Parma, Italy). This device consists of a disposable respiratory system comprising of a mouthpiece connected to a one-way aspiration valve and saliva trap with an EBC sample collection tube at the end inserted into a temperature controlled chilling unit set at − 5 °C. Due to the collection of exhaled air resulting in low sample volumes of EBC, sample collection employed 15 min of regular tidal breathing.

Immediately after collection of each EBC sample, an aliquot of EBC was diluted 10-fold with 0.5 mM EDTA (pH was adjusted to pH 8 using 10% v/v ammonia solution) onsite, to stabilise both Cr(VI) and Cr(III) and prevent interconversions of chromium species. This method of EBC collection and EDTA complexation is as previously described by Leese et al. (2017). The aliquot volume (µL) into EDTA and the remaining volume of EBC sample left in the collection tube were recorded. All samples were kept refrigerated at 2–8 °C after collection and complexation, during transportation to the laboratory and once at the laboratory until analysis.

### 2.3 Sample analysis

All EBC samples underwent speciation analysis to detect and quantify levels of Cr(VI) and Cr(III). The analysis was conducted in national laboratories from the participating countries. The laboratories applied their own analytical methods since a harmonised analytical method for all laboratories was not implemented. Each country analysed its own EBC samples, apart from The Netherlands whose EBC samples were analysed by the UK.

Speciation sample analysis was performed using a hyphenated liquid chromatography (LC), micro LC (µLC) or ion chromatography (IC) system with inductively coupled plasma – mass spectrometry (ICP-MS). The separation of Cr(VI) and Cr(III) was achieved using anion exchange columns. The analytical instrumentation used, chromatographic column and the limits of quantification (LOQ) achieved by each laboratory is stated in Table 1 (the calibration standard material, quality control material and the method of LOQ calculation for each countries analytical methodology is shown in Supplementary Table 1). All EBC samples were diluted 10-fold with EDTA as outlined in Section 2.2 above; no further dilution of the sample was performed and all were then injected directly into the analytical instrumentation.

**Table 1:** Analytical instrumentation used by each participating laboratory for the separation and measurement of Cr(VI) and Cr(III) in EBC samples, including the limits of quantification in µg/L.

	Analyte – Collision or reaction gas	Analytical Instrumentation	Anion Exchange Column	LOQ (µg/L)	
				Cr (VI)	Cr (III)
Finland	^52^Cr – He	1260 Infinity II HPLC (Agilent Technologies) coupled to a 7500 ICP-MS (Agilent Technologies)	Dionex IonPac AG7 (4 ×50 mm I.d.,)	0.003	0.003
France	^52^Cr - He	HPLC Advance (Bruker) with CombiPAL autosampler (CTC analytics) coupled to an Nexion 350X ICP-MS (Perkin Elmer)	Dionex IonPac AG7 (4 ×50 mm i.d.,) & AS7 (4 ×250 mm i.d.)	0.02	0.04
Italy	^52^Cr	Dionex ICS-5000 + coupled to an ICAP-Q ICP-MS (Thermo Fisher Scientific)	Dionex IonPac AG7 (2 × 50 mm i.d.) & AS7 (2 × 250 mm i.d.)	0.05	0.05
UK*	^52^Cr – 7% H_2_ in He	OneFAST µLC system (Elemental Scientific) coupled to an XSERIES2 ICP-MS (Thermo Fisher Scientific)	Dionex IonPac AG7 (4×50 mm i.d.,)	0.008	0.067

* The UK analysed EBC samples collected from the Netherlands. Therefore, the analytical methodology and LOQs outlined by the UK will also apply to the Netherland's EBC samples.

Although the analytical methodology for the measurement of Cr(VI) and Cr(III) in EBC samples was not harmonised across the participating countries through the HBM4EU Quality Assurance Scheme (Lopez et al., 2021), each analysing laboratory participated in an inter-laboratory comparison designed and implemented for this study. After an initial exploratory round, all laboratories were sent six samples, including a blank, spiked with Cr(VI) (QC3088, lot LRAB9395 – Sigma Aldrich, Dorset, UK) over the concentration range 0.134 – 3.36 µg/L (within the expected observed concentrations, based on previous work) (Leese et al., 2017). The four reporting laboratories showed an average recovery of 96.6% (range 83.9–113.1%, no concentration dependency observed) and an average variability of 8.2% (range 5.4–12.3%, concentration dependent as expected). No reporting laboratory failed to detect a spiked sample and all reported the blank as <LOQ (varied by laboratory but all <0.1 µg/L). When spikes of water and EBC were compared (most spikes were prepared in water due to difficulties in collecting sufficient EBC volume), the EBC sample showed better recovery (101.4% compared to 87.9%) and less variability (6.8% compared to 9.2%), giving confidence that real samples would reflect at least as good recovery and variability as the spiked samples.

### 2.4 Statistical analysis

Statistical analysis was performed using IBM© SPSS© statistical software suite (version 25/27, IBM Corporation, NY, US). Although the data is skewed (as is typical for biological samples), due to the relatively low number of samples the data was not log transformed and non-parametric tests (independent of the distribution) were used in the form of paired and unpaired tests such as Wilcoxon signed-rank test and Mann Whitney U test; p-values of < 0.05 were considered statistically significant. Where results less than the limit of quantification (LOQ) comprised more than 50% of the dataset for the occupationally exposed, statistical comparisons and correlations could not be performed. Descriptive statistics including means, median, range and percentiles levels (P95) were calculated using Microsoft Excel 2010 and box plots were prepared using Prism GraphPad software (version 6, SD, US). Values below the LOQ were substituted by LOQ/2 during the statistical processing (Hornung and Reed, 1990).

## 3 Results

### 3.1 Study results

In total 275 workers provided EBC samples in this study. There were 98 unexposed workers in the comparison control group providing a single EBC sample each. Of the 177 occupationally exposed workers, 343 EBC samples were collected. These consisted of 167 paired pre and post working week EBC samples and 9 unpaired single samples (2 workers providing a pre working week sample only and 7 workers provided a post working week sample only).

In the overall results published by Santonen et al. (2022), the occupationally exposed workers from the three target activities were further divided into seven new categories of more specific jobs within each of the target activities. This has not been done for the EBC results, primarily due to the reduced number of samples (only five of the nine countries participating in the HBM4EU chromates study collected EBC samples and not all workers in these countries provided EBC samples). Therefore, it should be noted that workers within the target activity of “chrome plating” will consist not only of chrome platers but other occupationally exposed workers working alongside them, for example machinists grinding or buffing chrome plated items. In addition, in the smallest group (surface treatment), the workers who provided an EBC samples consisted mainly of workers doing machining tasks and five thermal sprayers. It does not, therefore, inform on exposure during surface treatment by spraying or painting with chromate paint. (Table 2).

**Table 2:** Number of workers who provided EBC samples from each participating country to form the control group and the occupationally exposed group. The control group is split into numbers from Within Company and Outwith Company controls. The occupationally exposed group is split into numbers of workers from each of the three target activities.

	**Controls**			**Occupationally Exposed Workers**			
	**Total**	**Within Company**	**Outwith Company**	**Total**	**Chrome Platers**	**Welders**	**Surface Treatment**
**All countries**	**98**	**67**	**31**	**177**	**72**	**82**	**23**
**Finland**	25	9	16	33	13	15	5
**France**	22	22	0	57	21	18	18
**Italy**	25	25	0	33	0	33	0
**The Netherlands**	11	11	0	20	20	0	0
**UK**	15	0	15	34	18	16	0

**Fig. 1:**
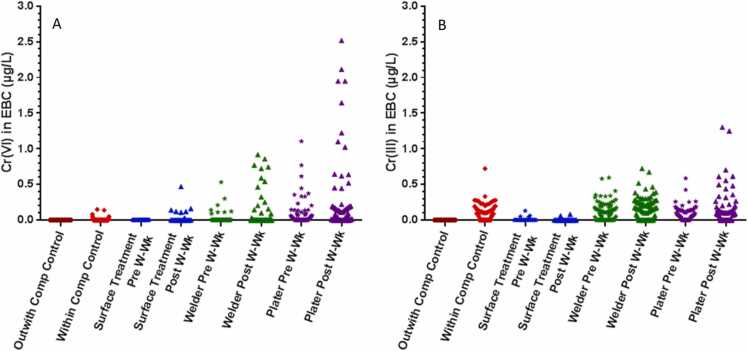
Scatter plots in µg/L from pre working week (Pre W-Wk) to post working week (Post W-Wk) for (A) Cr(VI) and (B) Cr(III) in EBC for occupationally exposed workers from each target group and the control group.

As presented in Table 3 the results for the analysis of the Outwith Company controls showed that 100% of the Cr(VI) and Cr(III) measurements were below the LOQ, compared with the results for the Within Company controls where only 87% and 34% of the EBC results were below the LOQ for Cr(VI) and Cr(III) respectively. The results indicate that the Within Company controls could have unintentional bystander exposure. Fig. 1 demonstrates the extent of this bystander exposure; their Cr(VI) exposure is higher than for the workers in surface treatment companies for pre working week results, and the Cr(III) exposure is higher than both the pre and post working week result for workers of the surface treatment target activity and is not dissimilar to the Cr(III) results from welders and chrome platers.

**Table 3:** Statistical summary of the range and 95th percentile (P95) in addition to the percentage of results less than the limit of quantification (LOQ) for Cr(VI) and Cr(III) in EBC samples from all the control group workers and when divided into control group categories Within Company controls and Outwith Company controls.

EBC samples	**Cr (VI) in EBC samples µg/L**			**Cr (III) in EBC samples µg/L**		
	**P95**	**Range**	**% below LOQ**	**P95**	**Range**	**% below LOQ**
All controls (**n = 98**)	0.05	<LOQ – 0.15	91%	0.27	<LOQ – 0.72	55%
1. Within Company (**n = 67**)	0.07	<LOQ – 0.15	87%	0.28	<LOQ – 0.72	34%
1. Outwith Company (**n = 31**)	< LOQ	< LOQ	100%	< LOQ	< LOQ	100%

The results for the EBC analysis from the occupationally exposed workers showed that 35% and 47% were below the LOQ for Cr(VI) and Cr(III) respectively. The results presented in Table 4 and in Fig. 1 show that in the “all workers” group the P95 for both Cr(VI) and Cr(III) in EBC samples increased from pre to post working week, indicating some occupational exposure through the working week.

**Table 4:** Statistical summary of the number, median, range and 95th percentile (P95) including percentage of samples less than the LOQ for pre working week samples (Pre W-Wk) and post working week samples (Post W-Wk) samples for Cr(VI) and Cr(III) in EBC in µg/L from occupationally exposed workers.

		**Cr (VI) in EBC (µg/L)**		**Cr (III) in EBC (µg/L)**	
		**Pre W-Wk**	**Post W-Wk**	**Pre W-Wk**	**Post W-Wk**
**All workers**	n = Median P95 Range %<LOQ	169 <LOQ 0.33 <LOQ – 1.10 72%	175 <LOQ 0.94 <LOQ – 2.52 57%	169 0.08 0.33 <LOQ – 0.59 36%	175 0.10 0.53 <LOQ – 1.30 37%
**Chrome Platers**	n = Median P95 Range %<LOQ	64 0.03 0.57 <LOQ – 1.10 41%	72 0.08 1.95 <LOQ – 2.52 33%	64 0.08 0.26 <LOQ – 0.59 27%	72 0.09 0.65 <LOQ – 1.30 33%
**Welders**	n = Median P95 Range %<LOQ	82 <LOQ 0.14 <LOQ – 0.53 88%	81 <LOQ 0.74 <LOQ – 0.92 74%	82 0.12 0.35 <LOQ – 0.60 30%	81 0.17 0.46 <LOQ – 0.72 25%
**Surface Treatment Workers**	n = Median P95 Range %<LOQ	23 <LOQ <LOQ <LOQ 100%	22 <LOQ 0.15 <LOQ – 0.47 68%	23 <LOQ 0.06 <LOQ – 0.13 83%	22 <LOQ 0.05 <LOQ – 0.08 91%

For Cr(VI), the chrome platers group had the highest pre and post working week median and P95. Although the overall Cr(VI) and Cr(III) results in EBC were lower for both welders and workers from surface treatment companies than the chrome platers, increases in levels of Cr(VI) from pre to post working week were observed for the P95 for both these target activities.

The median and P95 for Cr(III) in EBC increased from pre to post working week samples for both welders and chrome platers. Although the highest single Cr(III) result in EBC was from a chrome plater, the median Cr(III) was the highest in welders. The number of EBC results less than the LOQ for Cr(III) was very similar for welders and chrome platers (around 30%), unlike Cr(VI) where samples from welders were much more likely to be less than the LOQ than for chrome platers.

Table 5 shows a summary of the pre and post working week EBC results for Cr(VI), collected by each participating country for chrome platers and welders. However, to compare results from all countries, the data in Table 5 (only) has been adjusted to use the highest LOQ achieved for Cr(VI), (0.05 µg/L as shown in Table 1), rather than each analysing laboratory’s LOQ. Using the highest LOQ made little difference to the Cr(VI) results, however demonstrable exposure in welders in the UK could not be seen when using this higher LOQ.

**Table 5:** The number of samples, median, range and number of samples less than the LOQ for Cr(VI) in EBC for pre working week samples (Pre W-Wk) and post working week samples (Post W-Wk) of chrome platers and welders collected from each participating country, when adjusting the LOQ to the highest Cr(VI) LOQ reported, 0.05 µg/L.

**Cr (VI) in EBC µg/L**			**Finland**	**France**	**Italy**	**The** **Netherlands**	**UK**
**Chrome Platers**	Pre W-Wk	N = Median Range <LOQ	13 <LOQ <LOQ – 0.61 69%	21 <LOQ <LOQ – 0.14 81%	Not collected	12 0.02 <LOQ – 0.09 75%	18 0.14 <LOQ – 1.10 6%
	Post W-Wk	N = Median Range <LOQ	13 0.17 <LOQ – 2.52 8%	21 <LOQ <LOQ – 0.16 71%	Not collected	20 0.01 <LOQ – 0.22 80%	18 0.45 0.11 – 2.11 0%
**Welders**	Pre W-Wk	N = Median Range <LOQ	15 <LOQ <LOQ – 0.53 80%	18 <LOQ <LOQ 100%	33 <LOQ <LOQ – 0.30 85%	Not collected	16 <LOQ <LOQ 100%
	Post W-Wk	N = Median Range <LOQ	15 <LOQ <LOQ – 0.92 73%	18 <LOQ <LOQ 100%	33 <LOQ <LOQ – 0.33 88%	Not collected	16 0.03 <LOQ – 0.86 56%

Chrome platers had the highest Cr(VI) results in EBC samples collected by each participating country (Italy did not collect EBC samples from chrome platers). Like the results presented in Table 4, the median and the range also increased in Table 5 from pre to post working week for each participating country. The UK showed the highest median Cr(VI) results in EBC for both pre and post working week samples of chrome platers, Finland had the second highest Cr(VI) results. However, the range for both the UK and Finland were very similar. The UK was the only country to report all Cr(VI) results above the LOQ for the post working week EBC samples from chrome platers. Table 5 also presents Cr(VI) data for welders from each participating country (The Netherlands did not collect EBC samples from welders). All countries showed much lower Cr(VI) values for welders than for chrome platers. Finland showed the highest result for a pre working week Cr(VI) sample in welders of 0.53 µg/L and the highest post working week Cr(VI) of 0.92 µg/L. France reported “none detected” in all EBC samples collected from welders. There were insufficient EBC samples collected from workers within surface treatment companies (n = 23) to present the data in Table 5.

### 3.2 Statistical results

Statistical comparisons could not be performed on any of the EBC data from workers within surface treatment companies due to the low numbers of workers (n = 23) combined with the high number of samples less with results than the LOQ (Cr(VI) 84% and Cr(III) 87%. In addition, statistical analysis could not be performed for the Cr(VI) results in pre EBC samples from welders (81% less than the LOQ).

Mann Whitney statistical analyses showed there were statistically significant differences when comparing all controls and the results from chrome platers for both pre (p < 0.001) and post (p < 0.001) working week EBC samples for Cr(VI) and for both pre (p = 0.014) and post (p = 0.003) working week EBC samples for Cr(III). A statistically significant difference was also determined between all controls and Cr(III) in both pre (p = 0.001) and post (p < 0.001) working week EBC samples from welders.

Wilcoxon statistical analysis showed there was a high statistically significant difference between pre and post working week samples for Cr(VI) in EBC samples for chrome platers (p < 0.001) and for welders (p = 0.008). In addition, a significant difference was found between pre and post working week results for Cr(III) in EBC samples of welders (p = 0.004) and chrome platers (p = 0.044). No other statistical comparisons were determined where p = <0.05.

## 4 Discussion

A urine sample is currently considered the accepted matrix for biological monitoring of workers occupationally exposed to Cr(VI) (ACGIH, 2021; Hoet, 2005; HSE, 2020; Remy et al., 2021). The use of EBC analysis is a novel approach to determine specific Cr(VI) exposure, to enable the quantification of an internal dose of inhaled chromium. Although urinary total chromium remains a useful biomarker (Santonen et al., 2022), EBC offers an alternative, non-invasive, biological monitoring matrix, with speciation analysis that offers a low LOQ enabling the detection of low-level inhalation exposure to assist with occupational exposure interpretations as occupational exposure limits are further reduced. Therefore, the work presented here is another step forward to prove the applicability of EBC as a suitable biological sample for the biological monitoring of Cr(VI).

This study reports the results of Cr(VI) and Cr(III) concentrations in EBC samples collected from workers within three target activities: chrome plating, stainless steel welders and surface treatment workers. All levels of Cr(VI) and Cr(III) were below the LOQ for the Outwith Company control group, indicating no significant environmental or lifestyle confounders for this matrix. However, a key finding of the study was that this was not the case for EBC samples collected from the Within Company control group, indicating inadvertent exposure of bystander staff to Cr(VI). As reported by Santonen et al. (2022) in the wider HBM4EU study this trend can also be seen by the significantly higher urinary chromium levels in the Within Company controls compared to the Outwith Company controls. However, it is important to note that the EBC Outwith Company controls consisted of workers from only Finland and the UK, whereas the Within Company controls were from Finland, France, Italy and the Netherlands. Thus, contribution of country differences in background Cr(VI) exposure cannot be excluded. As shown in Table 4 and Fig. 1, Cr(III) in EBC samples of the Within Company control group are much higher than those of workers within surface treatment companies, and are more comparable to the levels of Cr(III) observed in stainless steel welders and chrome platers. This unintended exposure of non-chromium working staff was also reported in a UK study (Leese et al., 2017) where the (Outwith) control group had significantly lower Cr(VI) and Cr(III) levels in EBC samples than workers in administrative roles within the same companies as the Cr(VI) workers (equivalent to Within Company controls). This highlights an area for further hygiene input, of reviewing and improving housekeeping, including possible segregation of work areas. Smoking status and gender have little or no effect on internal chromium exposure (Santonen et al., 2022). This was also the case in earlier studies by Aprea et al., (2018); Hoet et al., (2013), Leese et al., (2017) and Morton et al., (2014).

In addition, the results reported here showed that the occupationally exposed worker group had higher levels of Cr(VI) in EBC than the control group. This was also true when the Cr(VI) results were organised into the activities: chrome platers and stainless steel welders (it must be remembered that the workers in the surface treatment target activity who provided an EBC sample consisted mostly of workers doing machining tasks and five thermal sprayers, so these results do not inform on exposure during surface treatment by spraying or painting with chromate paint). The chrome platers were found to have higher levels of Cr(VI) than welders and workers from within surface treatment companies. In addition, all four countries (that collected EBC samples from chrome platers) were in agreement that chrome platers had the highest levels of Cr(VI) in EBC samples.

It is important to note that within the wider HBM4EU study there were no specific target numbers for the enrolment of participants representing the three target activities. Meaning that the target activities sampled varied among the countries. This was dependant on the types of companies and workers willing to consent and participate in the study in each country. Thus the highest number of workers recruited was stainless steel welders, followed by chrome platers. The fewest workers recruited were from the target activity of surface treatment workers.

Previous studies looking at EBC samples as a biological monitoring tool to assess occupational exposure to chromium and reporting actual Cr(VI) results in EBC are few, but have been performed for chrome platers (Goldoni et al., 2006, Goldoni et al., 2010), various workers exposed to Cr(VI) including chrome platers (Leese et al., 2017) and stainless-steel welders (Riccelli et al., 2018). The Cr(VI) EBC results for welders reported in this study are much higher than those reported by Riccelli et al. (2018); although they collected EBC samples from 100 stainless steel welders, Cr(VI) could not be detected in any of the samples. The study by Goldoni et al. (2006) reported similar levels of Cr(VI) concentrations for chrome platers pre and post as is reported in our study. Although only 10 workers were sampled, they reported a decrease in Cr(VI) levels from overnight post shift to pre shift with levels ranging from 0.1 to 2.9 µg/L for post shift EBC samples and 0.1 – 2.1 µg/L for the following morning pre shift EBC samples. Alternatively, our study shows a majority increase in Cr(VI) concentrations from pre to post working week. Of the 64 chrome platers who provided a paired pre and post working week EBC sample in our study, 42 showed a post shift increase, 7 showed a decrease and the remaining 15 chrome platers had a pre and post result both less than the LOQ.

The finding that chrome platers showed the highest occupational exposure to Cr(VI) was demonstrated not only in EBC samples but also in both urine and blood samples that were collected and analysed as part of the wider HBM4EU chromates study (Santonen et al., 2022). This corroboration with urine and blood indicates that EBC could be a suitable medium to assess occupational exposures to Cr(VI). However, whilst the wider HBM4EU chromates study showed urine samples in welders to have higher levels than surface treatment workers, the RBC-chromium levels in welders showed the lowest occupational exposure to Cr(VI), this difference most likely reflects the different exposure aspects between workers in the different sectors and the different toxicokinetics of the chromium compounds they are exposed to. For example, it has been suggested that exposure by the inhalation of soluble Cr(VI) compounds does not translate to a higher risk of ill health effects beyond the lungs due to its rapid kinetics and elimination in urine (Remy et al., 2021). On the other hand, studies have intimated insoluble Cr(VI) compounds such as lead or strontium chromate found in paints and the ultrafine particles found in welding fume (Borthiry et al., 2008) are less likely to undergo reduction reactions once in the bloodstream (Abu Zeid et al., 2018, Afolaranmi and Grant, 2013), leading to persistent, longer-lived Cr(VI) species, which have the ability to then target other tissues and organs (Deng et al., 2019, Welling et al., 2015). However, as stated above, in the wider HBM4EU chromates study, elevated RBC-chromium levels in welders was not observed (Santonen et al., 2022), which was confirmed by the welders EBC results which showed elevated Cr(III) and very little Cr(VI) (as Cr(III) is unable to permeate cell membranes). The usefulness of EBC to assess occupational exposure could be dependent on the exposure pathway and the type of Cr(VI) compound used (such detail was not available from our study). For example, chrome platers are exposed to highly soluble chromic acid from the inhalation route (arising from the mists and vapours from the plating baths and tanks as well as contributions from potential skin absorption and ingestion from hand to mouth contamination) of the Cr(VI) solutions used in the plating baths and tanks. Whereas stainless steel welders are exposed to a complex mixture of welding fume that can contain low bioavailable, poorly soluble metallic chromium (Hedberg et al., 2010, Walter et al., 2015) that oxidises to Cr(VI) in the high temperatures used. However, incomplete oxidation of the metallic chromium often occurs (due to the lack of sufficiently high temperatures and time required) resulting in exposure to Cr(III) (Yu et al., 2014). Therefore, the expected inhalation and absorption of Cr(VI) for chrome platers will be higher than welders. For example, in the results of the wider HBM4EU chromates study (full data not reported here), although chrome platers showed higher chromium biomarker levels than other target activities, they did not have higher chromium air measurements than other target activities. The overall mean air levels for all workers were below 1 µg/m^3^. The mean Cr(VI) air level for chrome platers were 1.2 µg/m^3^ with only 16% of chrome platers wearing respiratory protective equipment (RPE) during plating bath operations. Whereas, the mean Cr(VI) air level was 1.6 µg/m^3^ for welders, who wore RPE over 50% of the time and surface treatment workers such as paint sprayers always wore RPE and their mean Cr(VI) air level was 12.5 µg/m^3^ (HBM4EU chromates study team, 2022, Viegas et al., 2022). This may indicate that the combination of the more soluble (and therefore more bioavailable) Cr(VI) the platers were exposed to and the lack of protection from RPE resulted in greater uptake, giving rise to the highest exposure as observed in biological sample results.

Both chrome platers and welders had significantly higher levels of both Cr(VI) (platers only p < 0.001) and Cr(III) (platers p = 0.014, welders p < 0.001) in their pre working week EBC samples than the Outwith Company control group, but as shown in Fig. 1 and Table 4 that whilst chrome platers had much higher Cr(VI) EBC levels, the welders had higher Cr(III) results for both pre and post working week than the chrome platers. This indicates the use of EBC sampling to assess Cr(VI) exposure is a more practicable vision for chrome platers than welders, but potentially it is also a useful exposure measurement for Cr(III) in welders.

The statistical analysis showed a lack of observed correlation between the levels of Cr(VI) or Cr(III) in EBC samples and the other sample matrices collected and analysed within the HBM4EU chromates study (urine, RBC, plasma, air samples and hand wipes (all p < 0.5)) (Santonen et al. (2022)). This lack of correlation applied to both EBC data of all workers and when the results were split into the target activities. An early study by Goldoni et al. (2006) of 10 chrome platers also found no correlation between total chromium in urine and Cr(VI) in EBC samples. The repeat study by Goldoni et al. (2010) and a study by Riccelli et al. (2018) again found no correlations between total chromium in urine and total chromium in EBC samples of 14 chrome platers and 100 stainless steel welders respectively. However, Goldoni et al. (2010) did observe a correlation (r = 0.57, *p* < 0.05) between total chromium in EBC and total chromium in RBC samples taken post working shift, but not with plasma samples. Furthermore, Caglieri et al. (2006) found a moderate correlation (r = 0.47, *p* < 0.05) between end of shift total chromium in EBC samples and total chromium of personal air samplers of 24 chrome platers. In the study presented here, one factor for the reduced statistical findings (and lack of correlations) could be explained by the high numbers of Cr(VI) and Cr(III) values below the LOQs making statistical significances and Pearson correlations unreliable. As shown in Table 1, the LOQ for Cr(VI) ranged from 0.004 µg/L up to 0.05 µg/L. It has been suggested that due to the kinetics of EBC production, biomarkers in EBC including chromium could have a dilution factor of approximately 1000 – 10000 (Goldoni et al., 2010), therefore, low limits of detection and quantification are critical for measuring Cr(VI) and Cr(III) in EBC samples.

The American Thoracic Society and European Respiratory Society Task Force has made a number of recommendations and factors for the consideration for the collection of EBC (Horvath et al., 2005, Horvath et al., 2017). Where relevant, the HBM4EU chromates study employed several of these recommendations to limit as much variation as possible during sample collection across the five participating countries to enable comparable findings of the data. The primary standardisation was the use of a single collection device amongst all countries called the TurboDECCS (Medivac A). All EBC collection devices are based on a freezing chamber, to cool and condense the exhaled breath, consisting of an inert material for the surface of the condensing chamber. Different devices use different inert materials and cooling temperatures. Other commercially available EBC collection devices are available such as the ECoScreen (or the modified version, ECoScreen-2) (Filt) or the RTube (Respiratory Research). Unfortunately, neither of the ECoScreen devices are portable, both being large and heavy bench-top instruments, and the RTube device, whilst portable, relies on aluminium sleeves (as the freezing cooling chamber) which need to be chilled or frozen prior to EBC collection and numerous sleeves are required for multiple EBC collections, meaning both were unsuitable for our occupational site visits. The TurboDeccs is portable and requires only a standard electrical connection to maintain the freezing cooling chamber at − 5 °C. Other recommendations that were employed were the environmental conditions of sample collection (for example, a suitable room away from the site of exposure, temperature controlled to general office conditions 20 – 25 °C) to minimise temperature and humidity variation. All workers were asked to breathe into the EBC collection device for 15 min, rinsing their mouths out with water beforehand to help remove food, saliva and other contamination from the mouth. Sample storage was also a standardisation factor, EBC samples were stored refrigerated between 2 and 8 °C after collection, during transportation, and at the analysing laboratory before analysis.

One known issue with EBC is the variable volume collected. The collection of EBC is non-invasive and does not cause an inflammatory response itself, however the collection of EBC results in low sample volumes. In this study all workers were asked to produce an EBC sample over a 15 min period, although the volume of EBC collected will vary from one individual to another. For example, in this study, the volume of EBC collected from each individual ranged from 131 to 4760 µL. This variability is due to the amount of air displaced in the lungs during normal inhalation and exhalation (tidal volume) and the amount of gas inhaled or exhaled from the lungs in one minute (minute of ventilation volume), which will affect the amount and size of airway lining fluid droplets (Gessner et al., 2001, Horvath et al., 2005, Liu and Thomas, 2007, McCafferty et al., 2004). However, the biggest variability to EBC volume is that these droplets of airway lining fluid are considerably diluted by the condensed water vapour in each EBC sample (Kuban and Foret, 2013). Unfortunately, unlike a urine sample where, for example, creatinine content can be measured to adjust for dilution, there has been no such dilution marker found to date for EBC. Some of the biomarkers investigated have been urea (Dwyer, 2004, Gessner et al., 2001), proteins (Gessner et al., 2001) and ion/electrolytes (Effros et al., 2002, Effros et al., 2003, Zacharasiewicz et al., 2004) although none have been proven to be definitive in providing a robust adjustment. Due to the correlation between tidal volume and minute of ventilation volume with EBC volume some have suggested that EBC results should be reported per volume of EBC collected (Gessner et al., 2001, Rosias, 2012) as a method to try and standardise EBC results. In the study presented here the total volume of EBC collected from each worker was measured. To determine if this form of dilution correction might be an appropriate way to present data, a statistical linear model could have been applied with EBC volume as a predictor variable; unfortunately due to the high number of results below the LOQ here, this was not possible. Therefore, the results of EBC volume collections has been withdrawn from the study. This variability and inability to volume correct EBC samples is likely to be the largest contributor to the reduced statistical findings and lack of correlations found in this study. The study by Caglieri et al. (2006) found no correlation with total volume of EBC and the levels of chromium and also withdrew EBC collection volume data from their study. A study by Winters et al. (2017) investigated a device (attached to the RTube EBC collection device) to assist the individual in regulating their breathing frequency to a set number of 10 breaths per minute and a breath volume of 1000 mL of air per breath to help reduce inter-subject and intra-subject EBC volume variability. Alternatively, several other studies have found a different method of dilution standardisation using a calculation involving the length of time taken to provide the EBC sample, the volume of EBC collected and the volume of exhaled breath, then expressing the results as the substance of interest exhaled in 100 L of exhalate (Knobloch et al., 2008, Reinhold and Knobloch, 2010, Reinhold et al., 2006). However, these studies used the ECoScreen (or the modified version, ECoScreen-2) EBC collection device, which allows the measurement of exhaled volumes, expiratory tidal volume, respiratory rates, expiratory minute ventilation and timed controlled collections. As previously discussed, ECoscreen are less suitable for occupational site visits due to their size. Moving forward however, since our chromate study was conducted, Medivac have developed the Volmet20 (Medivac B). The Volmet20 is a small device which connects to the disposable collection system and allows the user to input a specified volume of exhaled air in litres to be collected. Recording the time taken to reach this volume of exhaled air will allow the calculation of results in 100 L of exhalate, enabling the normalisation of samples as performed in the studies using the ECoScreen EBC collection device stated above.

For the future of trace elemental analysis in EBC it is important to fully understand the kinetics of EBC sample collection, for example the variability in the size and amount of droplets of airway lining fluid and the implications of water vapour dilution and how to correct for this dilution. However, until this time, it is anticipated this variability in findings will continue. Additionally, the toxicokinetics and toxicodynamics of chromium species in EBC also require investigation to enable a greater understanding of the dwell and excretion/elimination of chromium species following inhalation exposures, if EBC is to become a significant contributor to occupational exposure assessment.

One of the most important aims of the overall HBM4EU project is the harmonisation of methodologies and standardised collection of the data (Santonen et al., 2019, Galea et al., 2021). The project has focussed on laboratories’ performance in quality assurance/quality control (QA/QC) schemes designed for HBM4EU to demonstrate consistent comparable data rather than harmonisation of the analytical methods (Lopez et al., 2021). However, EBC being new research to HBM4EU, no QA/QC scheme was established, although an EBC inter-laboratory comparison was devised for the analytical partners as outlined in Section 2.1 of the Materials and Method. The differences in LOQ amongst the analysing laboratories (and the methods of calculation) have led to some incomparable data between the workers and different target activities. In future, multicentre studies such as this (and where analysis is pushing the measurement boundaries of low-level values), harmonising methods and establishing new and validated LODs and LOQs is crucial (despite the good agreement found in the interlaboratory comparison).

## 5 Conclusion

Although the kinetics are yet to be fully understood, the HBM4EU chromates study has shown that EBC has the potential to be a valid, non-invasive biological matrix to assess occupational exposure to Cr(VI) and Cr(III). Chrome platers exhibited the highest levels of Cr(VI) in EBC samples, which is in agreement with the findings from all biological media collected for the wider HBM4EU chromates study. Whilst exposures were low, chrome platers and welders were being exposed to Cr(VI) indicating that control measures in these target activities do not fully protect workers. In addition, Cr(III) was elevated in welders’ EBC samples implying this might be a useful biomarker worth future exploration. The study also demonstrated that EBC is still able to capture Cr(VI) exposure at mean air levels below 1.2 µg/m^3^, indicating EBC has a future as a sensitive biomonitoring method as more countries move to lower air limits. Further work to explore sample standardisation and to determine and fully understand the kinetics of EBC production and collection (in terms of sample dilution) and the toxicokinetics of chromium species in EBC, is needed to truly prove its potential as a suitable biomonitoring matrix to assess occupational exposure to Cr(VI).

## Funding

This project has received funding from the European Union’s Horizon 2020 research and innovation programme under grant agreement No 733032 and received co-funding from the author’s organizations and/or Ministries.

## Declaration of Competing Interest

The authors declare that they have no known competing financial interests or personal relationships that could have appeared to influence the work reported in this paper.

## Data Availability

The data that has been used is confidential.
