# Supplementary material for: HBM4EU chromates study – the measurement of hexavalent and trivalent chromium in exhaled breath condensate samples from occupationally exposed workers across Europe
Source: Toxicol Lett. 2023 Feb 15;375:59–68. doi: 10.1016/j.toxlet.2022.12.009 (PMC9887428; doi:10.1016/j.toxlet.2022.12.009)
Supplement: Supplementary file 1 — Supplementary material [file mmc1.pdf]

HORIZON2020 Programme
Contract No. 733032 HBM4EU

SOP 4:
Standard operating procedure for the collection of exhaled breath condensate samples

WP 8
Task 8.5

Table of Contents

Authors and Acknowledgements 2
1 Introduction 2
2 Exhaled Breath Condensate (EBC) 3
2.1 EBC collection devices 3
2.2 Complexation of EBC samples 4
2.3 Standardisation of EBC collection 4
2.4 Collection requirements 5
2.4.1 Equipment 5
2.4.2 Collection 5
2.4.3 Complexation with EDTA solution 7
2.5 Sample traceability 7
References 9

Authors and Acknowledgements

Lead authors:

This document has been developed by Dr Elizabeth Leese (PhD) and Kate Jones from the Health & Safety Laboratory (HSL).

Contributions were received from: Holger Koch (IPA) and Karen Galea (IOM).

This document has been created for the HBM4EU project. HBM4EU has received funding from the European Union's Horizon 2020 research and innovation programme under grant agreement No 733032.

1 Introduction

According to IARC, hexavalent chromium (Cr(VI)) compounds are classified as carcinogenic to humans (Group I) whereas trivalent chromium (Cr(III)) is an essential element.

In established biomonitoring (urinary total chromium), Cr(VI) exposure cannot be specifically determined because Cr(VI) compounds are reduced to Cr(III) in the body before being eliminated in urine. As exposures reduce due to increased demands from regulators, urinary total chromium methods will become less useful as it will be difficult to separate harmful Cr(VI) exposure from normal dietary Cr(III) exposure. In light of this, more specific biomarkers are being investigated; namely, Cr(VI) in exhaled breath condensate and in red blood cells (where it is trapped and isolated from Cr(III) as only Cr(VI) can enter the red blood cells but in the end it is not CrVI that is analyzed). This SOP covers the collection of exhaled breath condensate (EBC) samples for the analysis of Cr(VI); it is also possible to measure Cr(III) and possibly other metals in samples collected using this procedure.

2 Exhaled Breath Condensate (EBC)

EBC is a biological fluid which consists mainly of water vapour but also of small droplets of airway lining fluid from within the bronchial and alveoli regions of the lungs. These droplets of airway lining fluid contain an unknown fraction of both volatile and non-volatile substances but also environmental and occupational contaminants.

Inhalation is one of the primary routes of occupational exposure. It is thought that EBC might be a useful biological monitoring matrix when the current biological monitoring methods using traditional biological matrices such as urine or blood are not possible, or where the interpretation of elemental species in these matrices is difficult, for example hexavalent and trivalent chromium. Advancing the investigation of EBC may further the understanding of inhalation exposures and how elements behave and reside in the lungs.

The collection and analysis of EBC samples in this project is to further this understanding by gaining a more accurate picture of Cr(VI) exposure by correlating chromium species in EBC samples against industrial hygiene samples (for example personal air samples) in addition to urinary total chromium measurements.

The collection of EBC samples is an non-invasive technique and does not cause an inflammatory response itself. The collection of EBC results in low sample volumes and will take approximately 15 minutes to collect an adequate volume of sample (1-2 mL). Collection of EBC also involes the subsequent step of complexation with an EDTA solution to stabilise the hexavalent and trivalent chromium species immediately after collection.

The volume of EBC collected can vary from one individual to the next. EBC volume is directly correlated to tidal volume and minute/ventiliation volume. Tidal volume refers to the volume of air displaced in the lungs between normal inhalation and exhalation. Minute/ventilitation volume refers to the amount of gas inhaled or exhaled from an individuals lungs in one minute. Individuals with higher minute/ventiliation volume and/or higher tidal volume will produce more EBC[1-3]. This variation in EBC sample volume means the concentration of Cr(VI) will also vary. As there is currently no proposed volume correction marker (such as creatinine for urine) it is therefore suggested that EBC results must be reported in µg/L per volume of EBC collected.

2.1 EBC collection devices

All EBC collection devices are based on a freezing cooling chamber, to cool and condense the exhaled breath. This must consist of an inert material for the surface of the condensing cooling chamber such as glass, aluminium or Teflon as recommended by the American Thoracic Society/European Respiratory Society Task Force[1]. The effectiveness of EBC collection depends on the volume of breath passing into the condenser, the condensing surface area and the temperature gradient of the exhaled air to the cooling chamber[1].

A small number of commercial EBC devices are available to purchase for the collection of EBC. However, due to the differences in collection devices (for example the EBC collection temperature) only the TurboDECCS (Medivac SRL, Parma, Italy) system can be used to collect EBC samples as part of this project.

The TurboDECCS which stands for ‘Transportable Unit for Research of Biomarkers Obtained from Disposable Exhaled Condensate Collections Systems’, is a portable self contained thermoelectric peltier cooling device. An external power source is required to operate the Turbo, whose function is to cool the EBC collection tube (which is inserted into the cooling unit of the Turbo). The collection tube connects to the disposable EBC sampling kit comprising of a mouthpiece connected to a one-way aspiration valve with a saliva trap (DECCS). (See Figure 1 in section 2.4.1) The temperature for

the cooling chamber is -5°C (this is also the default temperature setting of the TurboDECCS so adjustment should not be necessary)..

A study by Goldoni et al[4] based on EBC collection volume, biomarker levels and collection variability determined the optimum EBC collection temperature of the TurboDECCS to be -5°C. Published studies using the TurboDECCS which focus on elemental concentrations in EBC also maintained this collection temperature of approximately 5°C[5-8].

It is not known how the differences in temperature and humidity of inspired air will affect the collection of EBC. A study by McCafferty et al[9] reported reduced EBC volume when individuals were inhaling cooler and drier air. For this project, EBC samples will be collected indoors in a standard office environment.

The volume of EBC collected can vary greatly however, 15 minutes of tidal breathing will collect on average between 1 – 2 mL of EBC. Several studies support the lack of correlation between EBC volume and gender, age, fitness, smoking status and lung status[2,3,9].

2.2 Complexation of EBC samples

Maintaining the stability and integrity of both Cr(VI) and Cr(III) within any sample matrix can be challenging, as the stability of both species is pH dependent. Generally Cr(VI) is stable in alkaline conditions whilst Cr(III) is stable in acidic conditions and forms Cr(III) hydroxide compounds in weak acidic conditions.

EBC is slightly acidic, with samples checked in earlier work at HSL having a pH between pH 6 and pH 6.5. Stability studies at HSL have shown that EBC samples analysed within 24 hours of collection which contain Cr(III) will elute the Cr(III) at two different retention times when not complexed with EDTA. It is HSL's thought that that the two Cr(III) peaks could be Cr(III) and the product of its slow conversion to a Cr(III) hydroxide.

Secondly as EBC samples and the EDTA solution are both slightly acidic, any Cr(VI) in the EBC samples will begin to slowly convert to Cr(III) if the EDTA solution is not adjusted to pH 8.

Therefore, to maintain the integrity of both chromium species it is impotant that each EBC sample is complexed with an EDTA solution immediately after collection.

A solution of 0.5 mM EDTA is made in water (using ultrapure deionised water – 18.2 MΩhm cm) and adjusted to pH 8 with 10% v/v ammonia solution. The EDTA complexes with Cr(III) and the adjustment of the pH to pH 8 stabilises Cr(VI).

To ensure that the same batch of EDTA solution prepared is used for both complexation of the EBC samples and for preparing standards and control material for the speciation analysis of those samples, a 2L EDTA solution is prepared. 1L can be taken to site to dilute with the EBC samples and the remaining 1L is used for the speciation analysis. The solution is kept at room temperature. Long term stability is unknown, as the solution was made fresh for each set of site visits/analysis in previous studies.

2.3 Standardisation of EBC collection

Although every volunteer will be asked to produce an EBC sample over 15 minutes, this will result in variable volumes of EBC. The variability is due to a result of the amount of air displaced in the lungs during normal inhalation and exhalation known as tidal volume and the amount of gas inhaled or exhaled from the lungs in one minute, known as minute of ventiliation volume. In addition, the droplets of airway lining fluid will be considerably diluted by the condensed water vapour in each

EBC sample, and this dilution will also vary considerably between volunteers. Unfortunately, unlike a urine sample where, for example, creatinine content can be measured to correct for dilution, there has been no such dilution marker proposed for EBC. Until a suitable biomarker of dilution correction can be found it is advised that volume (or weight) of EBC produced by each volunteer is recorded and that results are reported per volume of EBC. Due to the high content of water vapour in an EBC sample, 1 mL of EBC sample will weigh 1 g.

To help standardise the collection of EBC samples further, it is advised the volunteer rinse their mouth with water prior to providing a sample (drinking a cup of water is acceptable). This helps to remove any accumulated food and/or saliva from the mouth, helping to avoid any contamination of the EBC samples.

In addition, for the pre working week EBC samples, it is important that the volunteer has performed no practices or duties where Cr(VI) may occur.

2.4 Collection requirements

2.4.1 Equipment

1. Only the EBC collection device known as a TurboDECCS with disposable EBC sampling kits made of an inert material can be used. The temperature of the TurboDECCS should be at -5°C which is its default setting.
2. A suitable room away from the primary site of exposure/workshop floor (for example, office, meeting room, first aid/nurses room) with a operational plug socket (to power the EBC collection device) and a table and chair. For the comfort of the volunteer providing the EBC sample, a seated position with the collection device placed in front of them on a table is the most suitable.
3. Labels / or permanent marker pen
4. Nitrile disposable gloves or other suitable gloves
5. Secondary sample tubes with caps suitable for trace metal analysis. For example 15 mL - 50 mL polypropylene screw cap Sarstedt
6. Pipettes & tips (a 100 – 1000 µL, 20 – 200 µL and 500 – 5000 µL pipettes are the most suitable).
7. 0.5 mM EDTA solution adjusted to pH 8 with 10% v/v ammonia solution.
8. Drinking water & cups.

2.4.2 Collection

- A suitable room to collect EBC samples should already have been decided away from the primary site of exposure/workshop floor, for example office, meeting room or nurse/first aid room. To standardise the environmental conditions as much as possible ensure the room is within general office space conditions, for example a room temperature between 18-25°C.

  Plug the Turbo into a suitable power source that enables it to stand on a table where the EBC collection will take place. From room temperature it can take up to 20 minutes for the cooling chamber to reach -5°C.
- Wearing gloves unwrap and assemble a DECCS sampling kit and insert the EBC sample collection tube into the cooling chamber.
  - The DECCS sampling kits are individually wrapped and sealed and so will need opening and assembling according to the instructions available within each bag. For

hygiene and potential contamination reasons, each sampling kit is opened and assembled prior to each volunteer and not collectively beforehand.

  - For each subsequent EBC sampling kit, ensure the collection tube is inserted in the cooling chamber for at least 5 minutes prior to a volunteer beginning to provide their breath sample to allow the collection tube to cool.

- Ask the volunteer to rinse/wash their mouth out with water (depending on the facilities in the room, the volunteer can rinse their mouth by drinking the water or rinsing and spitting out the water) and then begin providing their breath sample by regular tidal (normal) breathing into the mouth piece for 15 minutes. For the most part a complete seal around the mouthpiece with the mouth and lips must be maintained, however periodic removal will be required to allow any accumulated saliva to be swallowed.
  - It may be advantageous to liken breathing into the mouthpiece to that of breathing with a snorkel or scuba equipment, and to breath through their mouth and not their nose. Remind them to keep their breathing tidal, as heavy or deep breathing may cause them to feel light headed.

- After 15 minutes and the volunteer has ceased providing their sample, remove the entire sampling kit from the Turbo unit, unscrew, cap and label the sample collection tube. Dispose of the remaining sampling kit, for example, in a biohazard bag.

- All samples are to be kept refrigerated at approximately 2-8°C after collection and complexation, during transportation to the laboratory and once at the laboratory until analysis, DO NOT FREEZE.

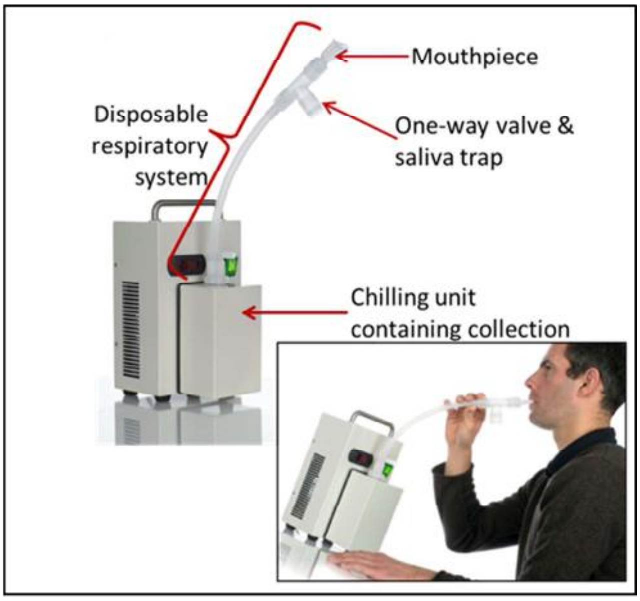

Please note that the disposable respiratory system (sampling kit) has been redesigned by medivac to be much shorter. The position of the volunteer to the mouthpiece is now much closer to the chilling unit than depicted in this photograph.

Figure 1. The Turbo-DECCS exhaled breath condensate collection device

If the Turbo is left without a collection tube inserted into the condensing cooling chamber for too long, the surface of the chamber will begin to form ice, prohibiting another collection tube from being inserted.

If a tube containing an EBC sample is left in the condensing cooling chamber for too long after the volunteer has ceased breathing into the sampling kit, the sample will begin to freeze. A frozen sample will deteriorate the integrity of Cr(VI).

2.4.3 Complexation with EDTA solution

Each individual will produce a different amount of EBC sample. It is therefore necessary to make a judgement as to what volume of EBC to use in the complexation with the pH adjusted EDTA solution. This is done on-site immediately after collection. Wearing gloves:

- Label a secondary sample tube and aliquot a suitable amount of the EBC sample into this tube. Note how much of the EBC has been transferred to enable accurate calculation of the volume of EBC collected for the reporting of the results.
- Dilute the aliquoted EBC sample 10-fold with the pH adjusted EDTA solution and cap.

An ideal scenario would be to aliquot 1 mL of EBC, so when diluted 10-fold with the EDTA solution it gives a final sample volume of 10 mL (ideally speciation analysis is performed in duplicate, and at least 1.5 mL is required per duplicate. This will leave the remaining complexed sample for any necessary repeats and if managanese & nickel analyses are required).

    - It is very possible a volunteer will produce less than 100 µl of EBC sample. This sample may not be suitable for analysis as a 10-fold dilution may produce an inadequate final volume for speciation analysis (determine minimum analytical volumes needed from your analysing laboratory).

- After complexation, place all EBC samples in a portable refrigeration unit/insulated box with ice pack until the samples arrive back at the analysing laboratory. DO NOT FREEZE.
- A short term storage study at HSL determined that these samples are stable for up to 6 weeks when stored refrigerated. It is not known how long the samples can be stored beyond this before Cr(VI) begins to deteriorate and convert.
- Upon returning to the laboratory, the remaining volume of EBC (uncomplexed) must be weighed and recorded. Each g of EBC correlates to 1 mL of EBC. To this weight, add (in g) the volume of EBC aliquoted for dilution with EDTA to give the original collected weight/volume of sample.

The collection pots are sealed within the sampling kits so cannot be weighed beforehand.

    - Centrifuge all the collection pots (to remove EBC from the side walls)
    - Weigh (g) and record an empty 30mL medicine beaker
    - Transfer the EBC sample to the 30mL medicine beaker and weigh (g) and record again.
    - Transfer back to the original container or another suitable container if retention of uncomplexed sample is required for other assays. Store appropriately for those assays.

2.5 Sample traceability

A standardised convention will be used to assign unique identification codes for all samples collected. The identification code convention is as follows:

Country ID (XX) - Participant ID (XX) - Sample ID (BXX/UXX/EXX/AXX/WXX)

Country ID 'XX' is the country code, using the ISO Alpha-2 country codes for the participating countries[1].

Country	ISO Alpha-2 country codes
Belgium	BE
Finland	FI
France	FR
Italy	IT
Poland	PL
Portugal	PT
The Netherlands	NL
United Kingdom	UK

Participant ID 'XX' is a two-digit running number of participants in each country (e.g. 01 for the first participant recruited, 02 the second and so forth).

Sample ID ‘UXX’ is one letter (B/U/E/A/W) to identify the type of sample collected, followed by a two-digit identifier (XX) to identify the running number of each type of sample for that worker (e.g. 01 for the first sample, 02 for the second and so forth). The letter code applied for the sample types is as follows:

Type of sample collected	Sample type code
Air	A
Blood	B
Exhaled breath	E
Urine	U
Wipe	W

The following scenario is provided to illustrate the application of this convention.

A worker is recruited in The Netherlands. He is the first worker recruited and is providing his first EBC sample. The sample identification code assigned is therefore:

NL-01-E01

In the event that an air sample is also collected from this same worker, the sample identification code to be assigned would be:

NL-01-A01

[1] http://www.nationsonline.org/oneworld/country_code_list.htm

References

1. I. Horvath, J. Hunt, P. J. Barnes, K. Alving, A. Antczak, E. Baraldi, G. Becher, W. J. van Beurden, M. Corradi, R. Dekhuijzen, R. A. Dweik, T. Dwyer, R. Effros, S. Erzurum, B. Gaston, C. Gessner, A. Greening, L. P. Ho, J. Hohlfeld, Q. Jobsis, D. Laskowski, S. Loukides, D. Marlin, P. Montuschi, A. C. Olin, A. E. Redington, P. Reinhold, E. L. van Rensen, I. Rubinstein, P. Silkoff, K. Toren, G. Vass, C. Vogelberg and H. Wirtz, *Eur. Respir. J*, 2005, **26**, 523-548.
2. J. Liu and P. S. Thomas, *Respiration*, 2007, **74**, 142-145.
3. C. Gessner, H. Kuhn, H. J. Seyfarth, H. Pankau, J. Winkler, J. Schauer and H. Wirtz, *Pneumologie (Stuttgart, Germany)*, 2001, **55**, 414-419.
4. M. Goldoni, A. Caglieri, R. Andreoli, D. Poli, P. Manini, M. V. Vettori, M. Corradi and A. Mutti, *BMC Pulm. Med.*, 2005, **5**, 10.
5. M. Goldoni, A. Caglieri, D. Poli, M. V. Vettori, M. Corradi, P. Apostoli and A. Mutti, *Anal. Chim. Acta*, 2006, **562**, 229-235.
6. A. Caglieri, M. Goldoni, O. Acampa, R. Andreoli, M. V. Vettori, M. Corradi, P. Apostoli and A. Mutti, *Environ. Health Perspect.*, 2006, **114**, 542-546.
7. M. Goldoni, A. Caglieri, G. De Palma, O. Acampa, P. Gergelova, M. Corradi, P. Apostoli and A. Mutti, *Journal of Environmental Monitoring*, 2010, **12**, 442-447.
8. S. Hulo, A. Radauceanu, N. Cherot-Kornobis, M. Howsam, V. Vacchina, B. de, V, D. Rousset, M. Grzebyk, M. Dziurla, A. Sobaszek and J. L. Edme, *Int. J. Hyg. Environ. Health*, 2016, **219**, 40-47.
9. J. B. McCafferty, T. A. Bradshaw, S. Tate, A. P. Greening and J. A. Innes, *Thorax*, 2004, **59**, 694-698.
