# Supplementary material for: HBM4EU chromates study – the measurement of hexavalent and trivalent chromium in exhaled breath condensate samples from occupationally exposed workers across Europe
Source: Toxicol Lett. 2023 Feb 15;375:59–68. doi: 10.1016/j.toxlet.2022.12.009 (PMC9887428; doi:10.1016/j.toxlet.2022.12.009)
Supplement: Supplementary file 2 — Supplementary material [file mmc2.docx]

|  | **Calibration Standard Material** | **Quality Control Material** | **Calculation of LOQ** | **Mobile Phase** |
| --- | --- | --- | --- | --- |
| **Finland** | Chromium(III) chloride hexahydrate (Sigma Aldrich) and Potassium dichromate 99% (Alfa Aesar) | In-house high and low Cr(VI) & Cr(III) standard spike in water | Half of the lowest calibration standard – 6 ng/L quantified with an RSD <20%. | 4% v/v ammonia solution & 3.2% v/v nitric acid pH adjusted to pH 2 |
| **France** | Ammonium dichromate, 99.999% metals basis (Alfa Aesar) and 1000 mg L single standard of Cr(III), PlasmaCAL (SCP Science) | HBM4EU Interlaboratory Cr ICI-EQUAS  &  In-house 1 µg L Cr(VI) and Cr(III) standard spike in water | Lowest concentration in EBC measured (taking into account the 10 fold dilution) quantified with an RSD <20%. Validated on 10 spiked samples | 60 mM ammonia nitrate adjusted to pH 8.3 |
| **Italy** | 100 mg L single standards of Cr(III) and Cr(VI) for ICP (CPAchem) | HBM4EU Interlaboratory Cr ICI-EQUAS  &  Proficiency testing material for Cr(VI) in drinking water | 10x the background equivalent concentration (BEC) of 10 independent speciation analyses | 0.25 M ammonium sulfate & 0.1 M ammonium hydroxide |
| **UK** | TraceCert 1000 mg L single standards Chromium (III) & (VI) for ICP (Sigma Aldrich) | Proficiency testing material for Cr(VI) in drinking water (QC1453, Lot LRAB7301) (Sigma Aldrich)  &  In-house 0.5 µg L Cr(VI) & Cr(III) standard spike in water | 10x the background equivalent concentration (BEC) of 22 independent speciation analyses | 4% v/v ammonia solution & 3.2% v/v nitric acid pH adjusted to between  pH 1.8 – 2 |

Supplementary Table 1
